# Fiber-Reinforced Plywood: Increased Performance with Less Raw Material

**DOI:** 10.3390/ma17133218

**Published:** 2024-07-01

**Authors:** Kristjan Saal, Heikko Kallakas, Eero Tuhkanen, Alar Just, Anti Rohumaa, Jaan Kers, Targo Kalamees, Rynno Lohmus

**Affiliations:** 1 Institute of Physics, University of Tartu, W Ostwaldi 1, 50090 Tartu, Estonia; kristjan.saal@ut.ee; 2 Laboratory of Wood Technology, Department of Material and Environmental Technology, Tallinn University of Technology, 19086 Tallinn, Estonia; heikko.kallakas@taltech.ee (H.K.); anti.rohumaa@taltech.ee (A.R.); jaan.kers@taltech.ee (J.K.); 3 Structural Engineering Research Group, Department of Civil Engineering and Architecture, Tallinn University of Technology, 19086 Tallinn, Estonia; eero.tuhkanen@taltech.ee (E.T.); alar.just@taltech.ee (A.J.); 4 Nearly Zero Energy Buildings Research Group, Department of Civil Engineering and Architecture, Tallinn University of Technology, 19086 Tallinn, Estonia; targo.kalamees@taltech.ee

**Keywords:** basalt fiber, plywood, reinforcement, layered structures, carbon footprint

## Abstract

Fiber-reinforced plywood is a composite material that combines the natural strength and rigidity of plywood with the added durability and resilience provided by reinforcing fibers. This type of plywood is designed to offer improved characteristics over standard plywood, including enhanced strength, stiffness, resistance to impact and moisture, and environmental degradation. By integrating reinforcing fibers, such as glass, carbon, or natural fibers (like flax, bamboo, or hemp) into or onto plywood, manufacturers can create a material that is better suited for applications where traditional plywood might fall short or when a decrease in product weight or savings in wood raw material are necessary. This report reviews the current progress in fiber-reinforced plywood in the context of plywood as a construction material to better understand the potential gains in plywood applications, mechanical parameters, and material savings. It is found that a simple and cost-effective procedure of fiber reinforcement allows for substantial improvements in plywood’s mechanical properties, typically to the extent of 10–40%. It is suggested that the wider adoption of fiber-reinforced plywood, especially in load- and impact-bearing applications, would greatly contribute to enhanced durability and longevity of the material while also allowing for more sustainable use of raw wood material.

## 1. Introduction

Plywood is a type of engineered wood product (EWP) manufactured from thin layers of wood veneer that are glued together with adjacent layers, with their wood grain rotated perpendicular to one another. It is an essential construction material that has a broad range of applications. In addition to traditional applications of wood, such as furniture and construction, plywood also finds extensive use in areas that benefit from its unique combination of toughness, lightweight, and low coefficient of thermal expansion, most notably as a building material for truck vans and LNG tanks for gas carriers. From a commercial perspective, plywood has a sizeable global market that was valued at USD 54.2 billion in 2022 and is projected to reach USD 73.3 billion by 2027 [1]. Nevertheless, these numbers come with a notable impact on the environment. Considering the average price of USD 350 per 1 m^3^ of plywood, the monetary global market in 2022 translates to ~155 million cubic meters of plywood, and considering that the overall yield from log to plywood is typically around 40–55%, i.e., the manufacture of 1 m^3^ of plywood requires up to 2.6 m^3^ of raw log material [2], this was ~400 million cubic meters in total log consumption. This, in turn, accounts for ~3 million ha in growing stock (i.e., an area equal to the size of Belgium), given the global growing stock averaging at 137 m^3^ per ha [3]. The world has a total forest area of 4.06 billion ha, which is 31 percent of the total land area, but more than half (54 percent) of the world’s forests are in only five countries—the Russian Federation, Brazil, Canada, the United States of America, and China [3]—meaning the availability of quality log material differs significantly around the globe. For example, in Europe, the total area of forests “clear-cut” harvested in 2016–2018 was 49% higher than in 2011–2015, according to a JRC study published in *Nature* [4]. However, the demand for plywood is increasing, and while there are no suitable alternatives in terms of material specifications and cost to compete with plywood effectively, so is the need for quality raw log material. In the list of recent green priorities put forward by many domestic and international regulations, preserving and restoring ecosystems and biodiversity stand out [5]. Better management of wood resources is one of them, and although plywood and other contemporary wood-based composites are a model of resource efficiency, they hold potential for further improvement in that category through fiber reinforcement.

From the perspective of sustainability, conventional plywood already tops the list of wooden construction materials. Life cycle assessment (LCA) is used to evaluate the environmental impact associated with every stage of a product’s life cycle. This includes the material extraction, processing, manufacturing, distribution, use, and disposal of materials, such as energy sources, water, and construction materials. Environmental impacts are typically assessed in various categories, such as carbon dioxide (CO_2_) emissions, energy consumption, water usage, and others and are often presented in metrics, such as kilograms of CO_2_ produced per unit of material mass. Costa et al. undertook a review of the life cycle assessment of wood-based panels and showed that plywood had the lowest environmental impact on average (331 kg CO_2_ eq/m^3^) [6]. Evidently, this is due to plywood’s unique layer-by-layer structure, which combines the strengths of a layer while diminishing its weaknesses. If the material is stronger and stiffer, it is possible to reduce its weight, which contributes to the LCA number and, consequently, the most sustainability. Nevertheless, plywood can be strengthened further by means of reinforcement (e.g., fibers) installed between the layers. Technologically simple and cost-effective, fiber reinforcement is extensively used in many conventional materials, such as concrete, plastics, etc. Unsurprisingly, the idea of bettering wood with fibers is not an exception or a recent discovery, with pioneering works on the matter dating back to the early 1980s [7]. Since then, a number of studies have been published unanimously demonstrating the potential of this methodology [8,9,10,11,12]. Despite this, fiber reinforcement has not been widely adopted by the plywood industry as of today, with the prevailing reason being that it has not provided the required benefit-to-cost ratio. Manufacturers would readily adopt fiber reinforcement if it could be seamlessly integrated into existing production lines in a cost-effective manner that ensured profitability. Additionally, the use of low-cost materials, such as polyvinyl acetate and basalt fiber, in the reinforcement process can further contribute to cost reductions [13]. This may not seem a serious challenge with so many studies out there pointing to the effectivity of fiber reinforcement and, consequently, to the obvious potential of utilizing less raw wood material for a given plywood product, but a closer look at the available data suggests it might not be minute either. First, plywood products are subjected to certain quality and dimensional prescriptions to which every innovation in technology must comply. Secondly, the existing studies on plywood reinforcement with fibers diverge greatly in terms of the methodologies used (i.e., the types and amounts of fibers used, the number of layers reinforced, and the parameters measured), making them very difficult to compare and thus, draw exhaustive conclusions for product development. Given the size of the task with the sheer number of different fibers and layouts potentially to be tested, it hardly makes sense to single out a certain combination or expect to effectively compare them for all relevant parameters. Instead, as a starting point, acquiring the context of plywood as a construction material is helpful.

## 2. Plywood as a Construction Material

A versatile and valued engineered wood product, plywood displays a diverse array of mechanical parameters that define its performance and suitability for various applications. Hardwood plywood is a commonly used material in various industries, and the selection of wood species significantly influences its properties. Birch and beech wood species have been extensively studied for their economic and technological properties in plywood manufacturing [14]. These species are prevalent in Europe and Nordic countries due to their favorable characteristics. Overall, plywood strength and stiffness, combined with its resilience and dimensional stability, make it an indispensable material in construction, even when compared with the strongest alternatives, such as steel. Moreover, compared to steel plates, plywood is considered more environmentally friendly, cost-efficient, and less prefabrication-demanding [15]. Compared to other wood products, plywood excels them all, being relatively homogeneous in terms of its strength and stiffness properties, i.e., even the lowest strength and stiffness properties of plywood are competitive with most of the structural timber made of softwood in a longitudinal (strongest) direction. Also, it exhibits decreased variability in mechanical properties compared to sawn timber [15].

**Figure 1:**
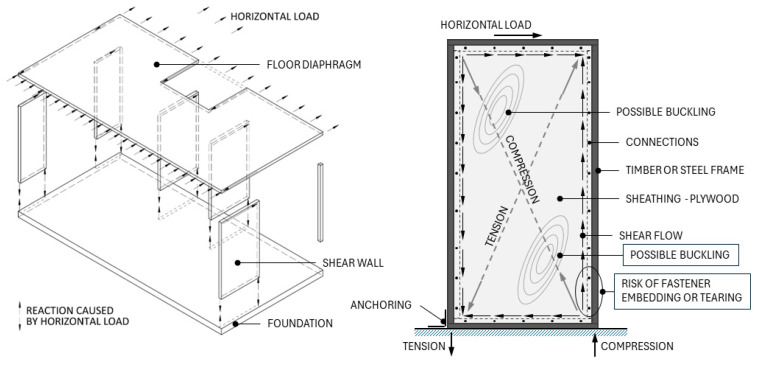
Left: Stiffening system principle of a building: floor diaphragm and shear walls [17]; right: forces act on the plywood sheathing in the frame structure; risk of plate buckling and fastener embedding or tearing.

In load-bearing structures, the use of plywood is most effective in places where high in-plane strength is required, which is the strongest property of this material. It is often used in stiffening structures of a building, where the horizontal load is transferred through the floor diaphragms and shear walls to the foundations (see Figure 1, left). Plywood fulfills its purpose as sheathing by providing the necessary stiffness and strength. Plywood is affected by shear force in the contour and compression and tension inside the frame (see Figure 1, right). The most significant limitation of using plywood in this situation is the buckling of the plate in the compression zone; the solution is to increase the plate thickness or add internal ribs to reduce the possible buckling area. Wang et al. showed that when loading plywood at an angle to the grain, the compressive and tensile strength decreases significantly at as early as 22.5 degrees and is weakest at 45 degrees [15]. Therefore, reinforcing plywood with diagonal reinforcement would create the possibility to increase the strength in the diagonal direction of the fibers and thus use the material more optimally. Another crucial place is the connection between the plate and the frame. If the fasteners have small edge distances, there may be a risk of the fastener tearing out from the edge, especially in the case of a connection with a steel frame. Local fiber reinforcement would reduce this risk, disperse stress concentrations, and further increase the embedment strength in the connection, resulting in a connection with a higher load-carrying capacity [16]. Song et al. evaluated the shear strength performance of cross-laminated timber connections and showed that using glass fiber-reinforced plywood in spline connections results in the best strength performance and a 1.7-times-higher yield strength than half-lapped connections that were designed according to the standards [16]. It is also useful for the industry as fiber reinforcement improves connection performance.

Wooden I-joists are efficient and optimized I-shaped engineering products, consisting of top and bottom flanges that resist bending and are combined with webs that offer shear resistance. Plywood was previously used as web material, but nowadays, OSB or particle board has replaced it. However, plywood can be used as a local web stiffener under highly concentrated loads [18]. It is common for there to be holes in the web for building communications (pipes, electricity, etc.). In such cases, reinforcement might be needed around the holes [19], which is a suitable application for plywood. With layered wooden engineering products (plywood and LVL), it is also possible to reinforce wooden and glulam beams by gluing them on the outside or as an intermediate layer or steel rod to resist high shear forces [20,21]. In such situations, it is important to keep the reinforcing plate as thin as possible so that it does not significantly increase the basic geometry of the member. Here, fiber reinforcement could be an opportunity to reduce the thickness of plywood.

The intermediate layer idea has also been applied to making dowelled timber connections with slotted-in steel plates, where the steel is replaced by compressed plywood and the steel dowels with fiber-reinforced polymer dowels [22]. While enhancing heat transfer in the connection area, metallic components often undermine fire resistance. Moreover, they limit the applicability of these connections in chemically harsh environments and with materials possessing non-magnetic and non-conductive properties [22]. Such a solution also has the potential to reduce the amount of steel in, for example, truss and frame systems and the associated carbon footprint for the reasons already stated above.

**Figure 2:**
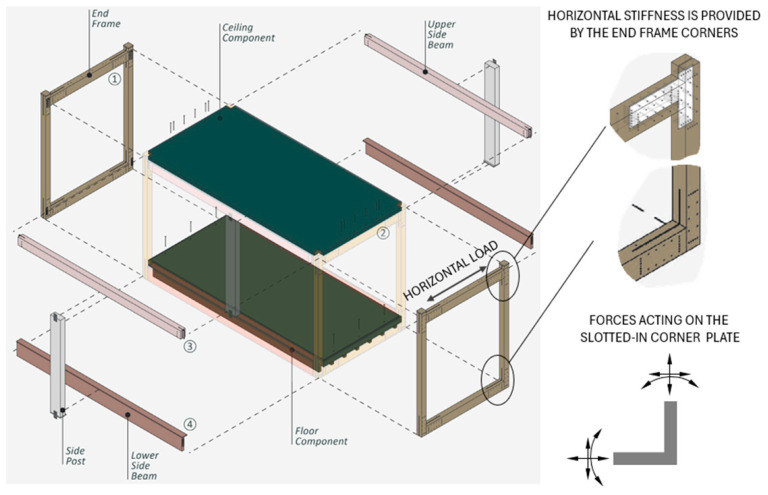
Example of moment-resisting frame based on “Pattern Building 369” system. [Figure is reproduced and modified from https://patternbuildings.com/downloads/ (accessed on 5 May 2024)].

Figure 2 shows the timber module of the “Pattern Building 369” system [23], where the end frames provide the transverse horizontal stiffness with moment-resisting corners. The original system uses slotted-in steel plates and self-drilling dowels. The purpose of the latter is to avoid the larger tolerances required when using conventional smooth dowels, where holes must be pre-drilled separately for wood and steel [24]. Larger tolerances reduce joint stiffness and efficiency. Replacing the steel plate with fiber-reinforced plywood would, in addition to the abovementioned advantages, allow easier installation of dowels because even though self-drilling dowels are designed to pass through both wood and steel, the practice has shown that this can be complicated, especially with higher grades of steel [17]. Mechanical advantages need to be further investigated. Direct comparative studies on steel and plywood in such connections are rare. In the early 1980s, ETH Zurich extensively studied timber trusses featuring steel-to-timber dowelled connections with multiple shear planes [25]. As part of this research, exploratory tests were carried out on timber-to-timber connections using internal plywood panels and hardwood dowels. The load-carrying capacity of these plywood connections was approximately two-thirds that of the trusses with internal steel plates and steel dowels, with the plywood panels failing and causing collapse.

**Figure 3:**
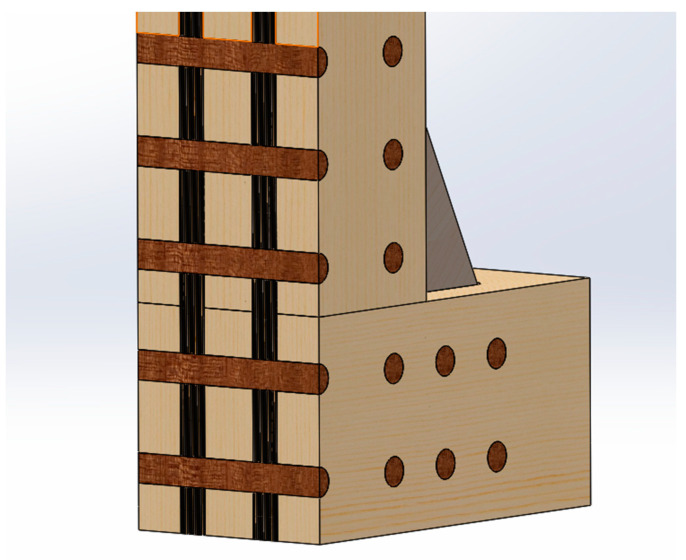
Possible solution for moment-resisting corner: slotted-in fiber-reinforced plywood with dowels.

An experimental study of the behavior of birch plywood gusset plates with mechanical connections [26] refers to the primary failure mode of net tension failure. However, it is a one-way tensile test; the plywood stress situation is even more complicated in the frame corner connection, but there is no comparative data on this with steel plates. The corner plate in the rigid frame is affected by a force changing in several directions simultaneously (in-plane bending and shear in two directions). Therefore, the plate must be homogeneous in its properties. Fiber reinforcement would make it possible to compensate for the weaker directions of the plywood and increase the embedment strength of the dowels. One possible corner solution with polymer (or steel) dowels is shown in Figure 3.

Fiber-reinforced plywood, when combined with wooden dowels, creates a robust connection that can effectively distribute loads and withstand stresses over time. These materials are compatible with timber, ensuring minimal differential movement and reducing the risk of joint failure due to environmental factors, such as moisture content fluctuations. Using materials that complement the properties of timber, such as wood dowels, the connection can achieve a higher level of integrity and longevity.

## 3. Fiber Reinforcement of Plywood

As can be seen from the above, plywood is used in applications that require substantial structural reliability. When plywood is reinforced with fibers, several mechanical properties can be significantly improved. The key properties that see enhancements include:

- Strength: fiber-reinforced plywood is more resistant to bending, tension, and compression forces [11,27,28,29,30];
- Stiffness: reduced deflection and increased overall structural integrity [31,32,33,34];
- Impact resistance: superior endurance of impacts and shocks [32,34,35];
- Fatigue resistance: increased durability under repeated stress cycles without failure [33,34];
- Creep resistance: enhanced ability to resist deformation over time under constant loads [35];
- Screw-withdrawal resistance: stronger resistance on the embedded screw upon withdrawal, making the fastenings more reliable [12,27,30,36,37];
- Moisture resistance and dimensional stability: while not a direct measure of mechanical strength, the addition of certain fiber types can improve plywood’s resistance to moisture and its dimensional stability, which are critical properties for many applications [31,36].

The degree to which fiber reinforcement improves the mechanical properties of plywood can vary widely, depending on several factors, including the type of fibers used (e.g., glass, carbon, natural), the method of incorporation (interleaving, surface bonding, or impregnation), the orientation of fibers, and the specific mechanical properties of interest (e.g., tensile strength, flexural strength, impact resistance). For reference, Table 1 shows some data for tensile strength and bending strength characteristics for fiber-reinforced plywood.

**Figure 4:**
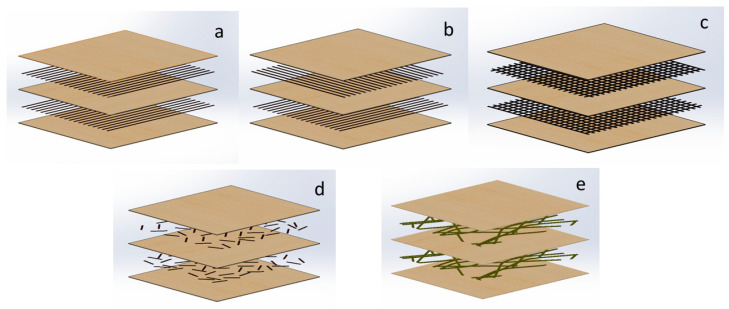
Possible fiber reinforcement schemes of plywood. (**a**) Parallel-aligned fiber layers are in the same direction to improve strength in one direction. (**b**) Parallel-aligned fiber layers are perpendicular for homogeneous load distribution. (**c**) Fiber mat solution for controlled technological applications. (**d**) Chopped fibers between veneer layers for large-scale applications. (**e**) Biological fibers between veneers for supporting energy-efficient product life cycle and waste management.

Several studies have looked into the effect of different types of fiber reinforcements on plywood characteristics (see Figure 4). Mostly, it is expected that synthetic fiber reinforcements can enhance the properties of plywood. Auriga et al. found that carbon fiber reinforcement increased both the modulus of rupture (MOR) and modulus of elasticity (MOE) of plywood [27]. Similarly, Bal et al. reported that glass fiber fabric reinforcement significantly increased the MOR and MOE of poplar plywood while reducing thickness swelling and water absorption [41]. Ashori et al. emphasized the benefits of fiber reinforcing with carbon fiber and waste rubber powder for improving mechanical properties, minimizing variability, and increasing durability [43]. Mei and Zhou also highlighted the reinforcement effect of glass fiber on poplar plywood, particularly when arranged close to the surface layer [44].

Further studies have been conducted on natural fiber reinforcements as an alternative to synthetic fibers. Moezzipour et al. demonstrated the effectiveness of natural fibers, such as kenaf, in improving the physical and mechanical properties of plywood [45]. Jorda et al. investigated the mechanical and physical properties of flax fiber-reinforced beech plywood, highlighting the importance of adhesive systems in optimizing its mechanical properties [12]. Lõhmus et al. studied how prestressing and temperature affect the tensile strength of basalt fiber-reinforced plywood [29]. They demonstrated how laminating pretensioned basalt fibers between veneer sheets can result in higher-strength plywood. This is congruent with the findings of Kramár and Král, who investigated the reinforcing effect of basalt fiber-reinforced polymer plywood coatings, displaying strength gains depending on the areal weight and position of the reinforcement [28]. Additionally, Kramár et al. investigated basalt fiber-reinforced polyvinyl acetate resin coatings for ductile plywood panels, indicating the potential for creating environmentally friendly reinforced composite wood-based panels [31].

Research has been conducted on investigating plywood reinforced with fibers and the effects of fiber alignment, shape, and sizes on plywood properties (See Figure 4). Auriga et al. showed that reinforcing plywood with carbon fibers increased the bending strength and modulus of elasticity, its perpendicular orientation improved the cutting force, and its core layer fibers reduced water uptake and thickness swelling [27]. Fiber length and orientation are also shown to affect the elasticity of fiber-reinforced plywood, emphasizing the importance of optimal fiber characteristics for achieving high reinforcement results. Xu et al. concluded that the random orientation of fibers leads to improved shear modulus and bending strength in plywood [46]. For the transport industry, it is possible to have improved buffering and toughening mechanisms with fiber mats in plywood [47]. Woven roving matts have shown better mechanical properties than chopped fibers in composites [48]. When it comes to composite materials reinforced with mats, it is possible to use chopped fibers and woven roving mats. In chopped-fiber mats, the fibers are randomly arranged and bonded with a binder. Chopped-strand mats have roughly similar mechanical properties in all directions. They are suitable for applications where conformability, cost-effectiveness, and resin absorption are critical considerations. Woven roving consists of long fibers woven into a grid-like structure. Woven roving provides high strength and stiffness in both directions. It is preferred when high strength and stiffness in both directions are essential. If uniform properties in all directions are needed, chopped-strand mats are a good choice. However, if high strength and stiffness, especially in both directions are required, woven roving is favored. Some research has shown that the combination of woven roving and chopped-strand mats in composites improves laminate composite properties by increasing the number of layers. In conclusion, the research consistently shows that fiber reinforcement in plywood, whether through materials like flax, basalt, carbon, or glass fibers, improves mechanical properties, durability, and strength performance. These studies collectively emphasize the significant role of fiber reinforcement in enhancing plywood properties, highlighting its importance in optimizing plywood performance.

Clearly, the length, diameter, and amount of loading (g/m^2^) of the fibers also affect the extent of reinforcement, as well as the laminating adhesive used. The quality and characteristics of the base plywood itself also play a significant role in the overall performance of the reinforced product. Combining the literature data, typically, fiber-reinforced plywood is about 10–40% stronger than normal plywood, depending on the aforementioned variables.

Concerning the potential gain in sustainability by fiber reinforcement, one has to bear in mind the type of fiber. Fibers, such as basalt, fiberglass, carbon fiber, and Kevlar, are commonly used to make high-quality fiber-reinforced plywood due to their strength and durability. However, these fibers do not exist naturally, meaning that energy must be spent to produce them. For example, the process of making basalt fibers involves several steps: mining and preparing the raw basalt rock, melting the basalt rock (at ~1400–1600 °C), extruding the fibers, the application of a sizing agent, cooling, and packaging. However, the overall energy cost must be balanced with the benefits these fibers offer. Typically, the fiber loading in fiber-reinforced plywood remains in the order of 50–200 g/m^2^ per layer, which is negligible in weight compared to that of the plywood panel itself. On the other hand, the sustainability aspiration can be greatly emphasized by using natural fibers, such as hemp, flax, coir, etc. The hemp and flax fiber industries are now growing, competing, and coexisting together with man-made fibers, particularly in the areas of quality, sustainability, economy, and production [49]. These fibers come in at a much lower processing cost than synthetic fibers, and in addition to being renewable and sustainable resources themselves, in some cases (e.g., coir), they are regarded as waste products that seek suitable applications. The good news is that performance-wise, natural fiber-reinforced plywood is fairly comparable to synthetic fiber-reinforced plywood. To illustrate this notion, Jorda et al. compared the mechanical properties of flax and glass fabric-reinforced birch plywood to unreinforced plywood, respectively, and found that every parameter studied (i.e., tensile strength, tensile shear strength, modulus of elasticity, modulus of rupture, and screw-withdrawal resistance) showed an increase for both flax and glass fabric with only minor variations [12]. Therefore, it is quite fair to assume that using natural fibers to manufacture fiber-reinforced plywood allows for more sustainability while comparing favorably with synthetic fiber-reinforced plywood in terms of mechanical properties, but if in pursuit of greater sustainability goals, plywood manufacturers should consider opting for natural fibers over synthetic ones. Last but not least, natural fibers have the additional advantage over synthetic fibers of perfect compatibility with existing plywood resins.

## 4. Importance of Adhesives

Commonly, veneers in plywood used for structural applications are bonded with petroleum-based adhesives. Currently, there is a growing focus on biobased structural adhesives, driven by the increasing emphasis on sustainability and environmentally friendly products. These adhesives are derived from renewable resources, such as plant-based materials, and they offer potential advantages over traditional petroleum-based adhesives in terms of reduced environmental impact and lower carbon footprint.

Natural polymers are primarily organic materials known for their diverse structures and multifunctional capabilities. As a result, they have found applications in various industrial contexts. While natural wood adhesives were historically utilized across numerous applications, they were eventually supplanted by synthetic alternatives. The renewed adoption of natural adhesives for wood bonding is still in its early stages. Key representatives of adhesives derived from natural resources include tannins, lignins, carbohydrates, unsaturated oils, proteins, protein hydrolysates, dissolved (liquefied) wood, and in situ generated adhesives, as observed in wood-welding self-adhesion or through surface activation [50,51,52].

However, the affordability and favorable adjustability of petroleum-based adhesives present challenges for introducing new biobased alternatives to the market. Sustainable adhesives must not only be cost-effective and biobased but also achieve satisfactory mechanical strength in manufactured panels; particularly, addressing moisture tolerance is a critical parameter that emerging biobased adhesives have yet to fully meet [53]. However, progress has already been made, and promising solutions have been found with lignin-based adhesive development solutions [30,54,55].

## 5. Conclusions

The fiber reinforcement of plywood offers a versatile and effective way to enhance its mechanical properties and performance, making it suitable for a wide range of demanding applications across various industries. The resulting composite material is better suited for structural applications where higher strength and rigidity are required. It is more resilient to external forces, such as impacts from heavy objects or mechanical loads. This is particularly beneficial in applications where durability and resistance to damage are essential, such as in construction, transportation, or marine industries. The addition of reinforcing fibers can improve the durability and longevity of plywood, making it more resistant to environmental factors, such as moisture, humidity, and temperature fluctuations. This can extend the lifespan of the material and reduce the need for frequent replacements or repairs. Furthermore, fiber reinforcement can allow for the production of lightweight plywood with comparable or even superior strength and stiffness compared to conventional plywood. This can be advantageous in applications where weight savings are critical, such as in the aerospace, automotive, or transportation industries. By adjusting the type, orientation, and volume fraction of reinforcing fibers, they can optimize characteristics such as strength, stiffness, flexibility, and impact resistance to suit the intended use, thus making it possible to tailor the properties of plywood to meet specific performance requirements for different applications. As for the more sustainable use of raw wood material, increasing the adoption of natural fiber-reinforced plywood in various applications would offer a great contribution to this purpose.

## Figures and Tables

**Table 1:** Some data for tensile strength and bending strength of fiber-reinforced plywood compared to unreinforced plywood.

Fiber Material	Tensile Strength	Bending Strength	Additional Comments
Basalt	+20–30% [38]	+15.7% [38] 10.4–99.5% [38]	Small addition of 10 wt.% total fibers led to a significant decrease in shrinkage and the coefficient of thermal expansion [33,34]. Strong increase in the strength-to-weight ratio [38]. Higher resistance to thermal degradation with the least amount of weight loss [33]. Basalt grids result in a higher bending strength without affecting internal bond strength [38]. Stiffness increases +9–42% [39].
Carbon	+17% [27]	37% [39] 20% [27]	The MOR increase varied between 57.6% and 102.9% [8].
Glass	+20% [40]	118% and 50% [41]	Glass fiber-reinforced plywood hardness increased by 36.4% [42].
Copper		+40% [32]	An increase in modulus of rupture was observed for all plywood made with the addition of copper fibers and veneer with holes [32].
Hemp		20% [30]	Natural hemp fiber and lignin–phenolic adhesives [11].
Kenaf		90% [10]	Use of reinforced adhesives [10].
Flax	+19% [11]		The behavior of cellulose fabric A is comparable to the flax fiber fabric [11].

## Data Availability

Not applicable.
